# The impact of metformin use on survival in kidney cancer patients with diabetes: a meta-analysis

**DOI:** 10.1007/s11255-017-1548-4

**Published:** 2017-03-07

**Authors:** Yang Li, Liyi Hu, Qinghong Xia, Yongqiang Yuan, Yonghua Mi

**Affiliations:** 1 Department of Nephrology, Yongchuan Hospital, Chongqing Medical University, Chongqing, 402160 China; 2 Department of Clinical Laboratory, Yongchuan Hospital, Chongqing Medical University, Chongqing, 402160 China

**Keywords:** Kidney cancer, Metformin, Prognosis, Hazard ratio (HR)

## Abstract

**Purpose:**

The effects of metformin on the prognosis of kidney cancer patients with diabetes are in controversial. The present study is conducted to classify the association of metformin use with the survival of patients with kidney cancer.

**Methods:**

Electronic databases, namely PubMed and Web of Science, were used to search the eligible studies up to December, 2016. Pooled hazard ratio (HR) and its corresponding 95% confidence interval (95% CI) were calculated. It was considered as statistically significant when *P* value was <0.05.

**Results:**

Eight cohorts were eligible for the present meta-analysis, including 254,329 kidney cancer patients. The combined HR suggested that the use of metformin could improve the overall survival (OS) (HR 0.643, 95% CI 0.520–0.795, *P* < 0.001) and cancer-specific survival (CSS) (HR 0.618, 95% CI 0.446–0.858, *P* = 0.004) in kidney cancer patients. In subgroup analysis, positive associations were found between metformin use and OS/CSS of localized renal cell carcinoma patients (OS: HR 0.634, 95% CI 0.440–0.913, *P* = 0.014; CSS: HR 0.476, 95% CI 0.295–0.768, *P* = 0.002). Moreover, we also found that the use of metformin could reduce the risk of death in kidney cancer patients (HR 0.711, 95% CI 0.562–0.899, *P* = 0.004).

**Conclusion:**

Our findings suggest that the use of metformin is in favor of the prognosis of patients with kidney cancers. Further investigations are needed to evaluate the prognostic value of metformin on kidney cancer patients.

## Introduction

Renal cell carcinoma (RCC) is recognized as the most common and lethal cancer of kidney cancer, accounting for 85% of all kidney cancers [1, 2]. What is more, the increasing incidence of kidney cancer is mostly attributed to the widely use of diagnostic imaging and the increasing rates of hypertension and obesity [3]. In addition, several studies have demonstrated that kidney cancer patients with diabetes mellitus have poorer survival compared with those without diabetes mellitus [4–6]. Another three articles also found that diabetes mellitus may be an independent risk factor for the survival of kidney cancer patients [7–9].

Metformin, a biguanide, is widely used for the therapy of diabetes with sulphonylureas [10]. Except for its use in diabetes, accumulating evidence has investigated that it could improve the survival of cancer patients including breast cancer, colorectal cancer, and prostate cancer patients [11–13]. However, its effects on the survival of kidney cancer remain unclear. The articles reported by Nayan et al. [14, 15] showed that the use of metformin was not associated with the overall survival (OS), cancer-specific survival (CSS), and disease-free survival (DFS) of RCC patients. However, Keizman et al. [16] found that RCC patients treated with metformin have lower risk of mortality than those without metformin therapy. In addition, another study found that compared to metformin non-users, metformin use on RCC patients was in favor of better CSS and DFS [17]. Thus, the impact of metformin on survival outcomes of patients with kidney cancer was in controversial.

To obtain a more comprehensive estimation of the prognostic significance of metformin in RCC patients, we performed the present meta-analysis to explore the effects of metformin on OS, CSS, DFS, and PFS of patients with RCC.

## Materials and methods

### Retrieval of studies

We performed a comprehensive search of two electronic databases, namely PubMed and Web of sciences (up to November 2016). The search strategy was based on Mesh headings, key words and text words as follows: “metformin” combined with “kidney cancer” or “kidney carcinoma” or “kidney neoplasm” or “renal cancer” or “renal carcinoma” or “renal neoplasm” or “renal cell carcinoma”. In addition, the references listed in the retrieved studies were also reviewed.

### Selection of the eligible studies

We firstly comprehensively screened the titles and abstracts of the candidate articles. Then, the full text was reviewed to exclude the articles which could not be eliminated at the initial screening of the titles and abstracts.

The criteria for eligible studies were defined as: (1) articles published in English; (2) original studies, not review or meta-analysis; (3) patients diagnosed as kidney cancer patients with diabetes; (4) articles estimating the association between metformin use and survival of kidney cancer patients; (5) eligible data could be obtained including hazard ratio (HR) and 95% confidence interval (95% CI); exclusion criteria: (1) letters, reviews, and articles not published; (2) articles without the eligible data of either HR or 95% CI.

### Data extraction

If available, the following items were extracted: name of first author, country, population, publication date, mean or median age, follow-up time (mean or median months), total number of patients, amount of male patients, number of patients received surgery therapy, counts of metformin users and metformin non-users, pathological type of kidney cancer (localized RCC or metastatic RCC), HR and 95% CI of the patients’ survival outcomes.

### Statistical analysis

Pooled HR and its corresponding 95% CI were used to evaluate the association of metformin use with OS, PFS, DFS, and CSS of kidney cancer. It suggested poor prognosis when HR was larger than 1 and the corresponding 95% CI did not overlap 1. *Q*-tests and *I*-squared test were used to assess the statistical heterogeneity of studies. It was considered as no obvious heterogeneity when *P* ≥ 0.05 or *I* ^2^ ≤ 50%. We performed sensitivity analysis by sequentially omitting individual studies when there was statistically heterogeneity. Publication bias was only performed for meta-analysis involving more than five studies via Begg’s test and Egger’s test. STATA 11.0 was used to analyze the results, and it was considered as statistically significant when *P* < 0.05.

## Results

### Main characteristics of the included studies

**Fig. 1:**
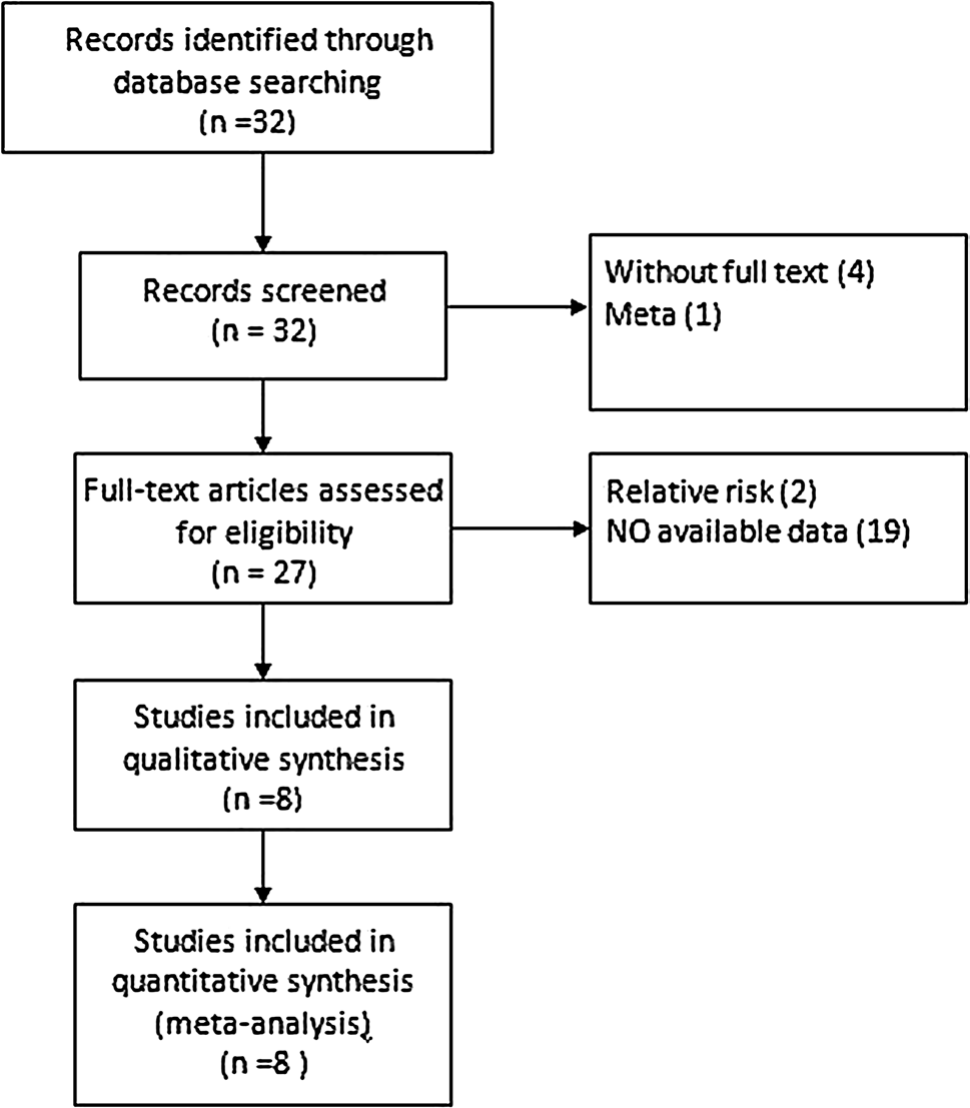
The procedure of the search strategy

Thirty-two articles were identified in our meta-analysis, of which 18 articles were eliminated at the initial screening of the title and abstract. Finally, eight eligible publications [1, 14–20] were included after comprehensively reviewing the full text. Detailed search strategy is shown in Fig. 1. The main characteristics of the eligible studies for metformin use on kidney cancer patients are summarized in Tables 1 and 2. A total of 254,329 patients were included, of which male patients accounted for 52.5%. In addition, there were four studies on localized RCC [14, 15, 19, 20], two studies on metastatic RCC [16, 18] and one study both on localized and metastatic RCC [17].

**Table 1:** Main characteristics of the eligible studies in this meta-analysis

Author	Year	Country	Population	*N*	Age	Male	Follow-up time (months)	Surgery or radiofrequency ablation	Metformin users/non-users	Pathological type	All cause death	KC-specific death
Nayan et al.	2016	Canada	North American	613	77.9^a^	367 (59.9%)	272 (44.4%)	NR	Localized RCC	409	194 (31.6%)	
Hamieh et al.	2016	New York	North American	4736	61.5^b^	3363 (71%)	18.04^b^	3325 (70%)	218/4337	Metastatic RCC	NR	NR
Nayan et al.	2016	Canada	North American	158	63.9^b^	113 (71.5%)	43^b^	158 (100%)	82/76	Localized RCC	NR	NR
Keizman et al.	2016	Israel	Asian	108	66.5^a^	70 (64.8%)	25^a^	92 (85.2%)	52/56	Metastatic RCC	NR	NR
Cheng et al.	2016	Singapore	Asian	290	59.4^a^	131 (45.2%)	59.1^a^	277 (95.5%)	131/159	Localized RCC	NR	33 (11.4%)
Cheng et al.	2016	Singapore	Asian	100	61.9^a^	72 (72%)	8.8^b^	41 (41%)	53/47	Metastatic RCC	NR	79 (79%)
Psutka et al.	2014	New York	North American	283	67^b^	88 (31.1%)	97.2^b^	283 (100%)	83/200	Localized RCC	NR	NR
Hakimi et al.	2013	New York	North American	784	62^b^	549 (70%)	43.2^b^		55/729	Localized RCC	NR	NR
Tseng et al.	2015	Taiwan	Asian	247,252	NR	129,052 (52.2%)	44,831 (18.1%)	171,753/75,499	NR	1741	NR	

^a^Mean, ^b^ Median, *NR* not reported

**Table 2:** Survival data on prognosis of the included studies in this meta-analysis

Author	Survival analysis	Univariate HR (95% CI)	*P* value	Multivariate HR (95% CI)
Nayan et al.	OS, CSS	OS: 0.99 (0.78–1.25)		OS: 1.09 (0.85–1.40)
		CSS: 1.12 (0.80–1.55)		CSS: 1.25 (0.88–1.77)
Hamieh et al.	OS, PFS	OS: 0.771 (0.566–1.049)^a^	0.098	
		OS: 1.053 (0.837–1.324)^b^	0.6606	
		PFS: 0.905 (0.682–1.199)^a^	0.4858	
		PFS: 0.979 (0.806–1.189)^b^	0.8274	
Nayan et al.	OS, CSS, DFS	OS: 0.86 (0.40–1.85)	0.07	
		CSS: 0.38 (0.08–1.86)	0.23	
		DFS: 0.99 (0.36–2.74)	0.98	
Keizman et al.	OS, PFS	OS: 0.42 (0.26–0.69)	0.001	
		PFS: 0.71 (0.47–1.08)	1	
Cheng et al.	CSS, DFS	CSS: 0.23 (0.09–0.61)	0.0028	
		DFS: 0.47 (0.27–0.81)	0.0058	
Cheng et al.	CSS	CSS: 0.78 (0.50–1.23)	0.29	
Psutka et al.	OS, CSS, DFS	OS: 0.58 (0.38–0.87)	0.01	
		CSS: 0.62 (0.32–1.21)	0.16	
		DFS: 0.87 (0.51–1.48)	0.6	
Hakimi et al.	CSS, DFS	CSS: 0.76 (0.21–2.70)	0.7	
		DFS: 1.02 (0.59–1.74)	1	DFS: 1.22 (0.66–2.27)
Tseng et al.	OS	OS: 0.279 (0.254–0.307)	<0.0001	

^a^Metformin users versus other antidiabetic therapy

^b^Metformin users versus antidiabetic therapy non-users

### Outcomes of all-caused mortality

**Fig. 2:**
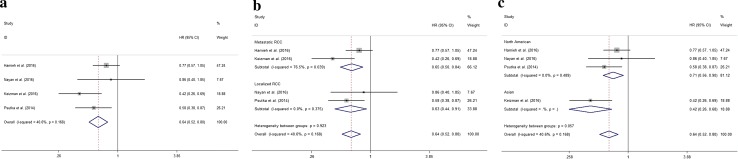
Association between metformin exposure and OS of kidney cancer patients. **a** All studies with excluding the source of heterogeneity; subgroup analysis, **b** studies related with localized RCC, **c** studies associated with North American patients

The association of metformin use with OS of kidney cancer patients was reported in six articles involving 253,150 patients [1, 14–16, 18, 19], three localized RCC studies (1054 patients) [14, 15, 19] and two metastatic RCC studies (4484 patients) [16, 18]. The pooled HR showed that compared to patients without being exposed to metformin, a reduced risk of mortality was observed in patients exposed to metformin [HR (95% CI) 0.41 (0.38–0.45), *P* < 0.001]. However, there was significant heterogeneity among these studies (*P* < 0.001, *I* ^2^ = 97.1%). Although we performed subgroup analysis, it was difficult to find the source of heterogeneity. Then we conducted sensitivity analysis and found the source of heterogeneity was from the studies reported by Nayan et al. [14], Hamieh et al. [18] and Tseng et al. [1] which composed of much more patients than the rest. The summary HR (95% CI) was changed to 0.643 (0.520–0.795) (*I* ^2^ = 40.6%, *P*_heterogeneity_ = 0.168; *Z* = 4.08, *P* < 0.001, Fig. 2a) after exclusion of the studies contributed to the heterogeneity. In addition, subgroup analyses were used to identify the different influences of metformin use on populations from different regions of the world and patients with or without metastases. We found a significant reduced risk of death in localized RCC patients exposed to metformin [HR (95% CI):0.634 (0.440–0.913), *I* ^2^ = 0.00%, *P*_heterogeneity_ = 0.375; *Z* = 2.45, *P* = 0.014, Fig. 2b]. The results also showed that the use of metformin was in favor of weakening the risk of death for North American RCC patients [HR (95% CI) 0.711 (0.562–0.899), *I* ^2^ = 0.00%, *P*_heterogeneity_ = 0.489; *Z* = 2.84, *P* = 0.004, Fig. 2c]. Although we observed the same result in RCC patients with metastasis, there was significant heterogeneity [HR (95% CI) 0.648 (0.449–0.841), *I* ^2^ = 76.5%, *P*_heterogeneity_ = 0.039; *Z* = 3.26, *P* = 0.001].

### Outcomes of kidney cancer-specific survival

**Fig. 3:**
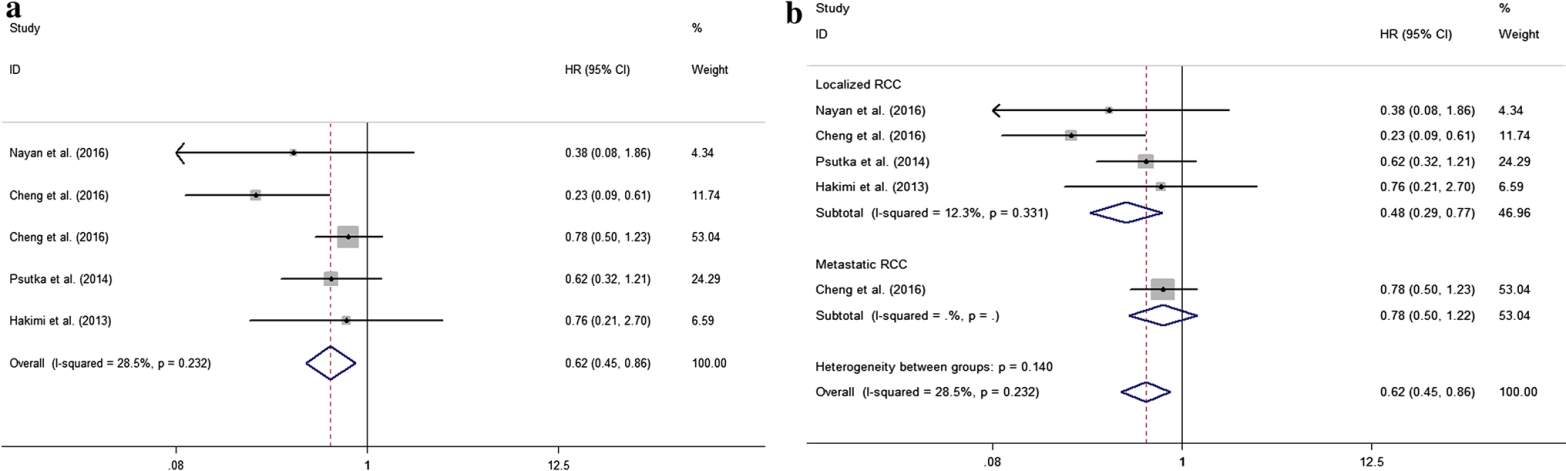
Relationship of metformin exposure with CSS of kidney cancer patients. **a** All studies with omission of the studies contributed to the heterogeneity; subgroup analysis, **b** studies in relation to localized RCC

The relationship of metformin use with kidney cancer-specific mortality was studied in five articles recruiting 2228 patients [14, 15, 17, 19, 20], four studies [14, 15, 19, 20] on localized RCC (1838 patients) and one study [17] both on localized and metastatic RCC (290 localized RCC patients and 100 metastatic patients). Although the pooled HR suggested that no association was observed between CSS and patients exposed to metformin or not, there was a high degree of heterogeneity [HR (95% CI) 0.830 (0.658–0.1.048), *I* ^2^ = 57.8%, *P*_heterogeneity_ = 0.037; *Z* = 1.57, *P* = 0.117]. In the sensitivity analysis, we found the study reported by Nayan et al. [14] contributed to the heterogeneity. There was no significant heterogeneity after the omission of the study published by Nanyan et al. [14] And the risk of kidney cancer specific due to kidney carcinoma was decreased in patients treated with metformin in comparison with the patients without use of metformin [HR (95% CI) 0.618 (0.446–0.858), *I* ^2^ = 28.5%, *P*_heterogeneity_ = 0.232; *Z* = 2.87, *P* = 0.004, Fig. 3a]. Moreover, in the subgroup analysis, the use of metformin reduced the risk of death in patients with localized RCC patients [HR (95% CI) 0.476 (0.295–0.768), *I* ^2^ = 12.30%, *P*_heterogeneity_ = 0.331; *Z* = 3.04; *P* = 0.002, Fig. 3b]. No associations were found between the use of metformin and the North American populations with RCC [HR (95% CI) 0.606 (0.349–1.053), *I* ^2^ = 0.00%, *P*_heterogeneity_ = 0.293; *Z* = 1.78; *P* = 0.076].

### Outcomes of DFS and PFS

In the present study, two articles were on PFS (4844 metastatic RCC patients) [16, 18] and four articles were on DFS (1515 localized RCC patients) [15, 17, 19, 20]. The pooled HRs showed that there was no associations between metformin use and PFS/DFS of RCC patients [PFS: 0.919 (0.791–1.067), *I* ^2^ = 0.00%, *P*_heterogeneity_ = 0.388, *Z* = 1.11, *P* = 0.266; DFS: *I* ^2^ = 34.5%, *P*_heterogeneity_ = 0.205, *Z* = 1.72; *P* = 0.086].

### Publication bias

The Begg’s test and Egger’s test were used to evaluate the publication bias in meta-analysis. The results of Begg’s test and Egger’s test revealed no obvious publication bias with considering the existence of heterogeneity or not [OS: *P*_begg’s_ = 0.548, *P*_egger’s_ = 0.097 (with heterogeneity); CSS: *P*_begg’s_ = 0.260, *P*_egger’s_ = 0.080 (with heterogeneity) CSS: *P*_begg’s_ = 0.73, *P*_egger’s_ = 0.307 (without heterogeneity)].

## Discussion

### Key findings

The present meta-analysis is the first to discover the association between metformin and kidney cancer patients. The results showed that metformin could improve the OS [HR (95% CI) 0.643 (0.520–0.795), *P* < 0.001] and CSS [HR (95% CI) 0.618 (0.446–0.858), *P* = .0004] of patients with kidney cancer. In the subgroup analysis, we also found that in kidney cancer patients, metformin users could have better prognosis (OS) than metformin non-users. In addition, the subgroup analysis also suggested that compared to patients treated without metformin, OS and CSS were both improved in localized RCC patients treated with metformin, which was not found in metastatic RCC. Nevertheless, our current meta-analysis did not show association of metformin with the DFS and PFS of kidney cancer.

### Comparison with other studies

The results of our meta-analysis on OS and CSS were in consistent with the previous studies. It has been reported by Tseng et al. [1] that better OS was associated with the use of metformin in kidney cancer patients. Moreover, Keizman et al. [16] and Psutka et al. [19], respectively, found in metastatic and localized RCC that the risk of mortality was reduced in cancer patients. Up to data, only one study related to localized RCC demonstrated that metformin could decrease the risk of kidney cancer-specific death in RCC patients [17]. However, another three articles identified that metformin showed no association with the OS of both localized and metastatic RCC patients [14, 15, 18]. Meanwhile, four researches associated with localized and metastatic RCC suggested that the use of metformin had no effects on the CSS of patients with RCC [14, 17, 19, 20]. In addition, among five studies on DFS, only one study published by Cheng et al. [17] showed positive association between metformin use and the DFS of localized RCC patients, which was in contrast to our findings. Additionally, two studies on PFS found that there were no effects of metformin on the PFS of metastatic RCC patients [16, 18].

### Strengthens and weakness

We are the first to explore the influence of metformin on the prognosis of kidney cancer patients with diabetes. And in this meta-analysis, localized RCC and metastatic RCC are both included. Moreover, we also conducted subgroup analysis to identify the different effects of metformin on localized and metastatic RCC. However, there are limitations in our meta-analysis. The sample size and population differ in different articles included in this meta-analysis, which may contribute to the heterogeneity and affect the outcomes of this meta-analysis. As shown in Table 1, the sample size of the article reported by Tseng et al. [1] is much larger than other articles and the patients included in this study are Asian, which contribute to the heterogeneity of this meta-analysis and play a great role in identifying the influences of metformin on the prognosis of Asian kidney cancer patients. The subgroup analysis in Fig. 2c also has shown that only one article reported the effects of metformin on the OS of Asian kidney cancer patients. Therefore, further studies are needed to explore the association between metformin use and various populations to exclude the heterogeneity. And the patients of the eligible studies are mostly diagnosed as localized RCC, which makes it difficult to investigate the association between the use of metformin and outcomes of metastatic RCC patients. In addition, the clinical information extracted from the eligible studies were limited, which makes it difficult to make further subgroup analysis to analyzed whether the impacts of metformin on kidney cancer patients are related with age, sex, and other characteristics.

## Conclusion

The current meta-analysis suggests that the use of metformin could improve the OS and CSS of kidney cancer patients, especially localized RCC patients. However, due to the limitations of this meta-analysis, further investigations are needed to be conducted to identify the different effects of metformin on patients with localized and metastatic RCC.
